# The Hemianopia Reading Questionnaire (HRQ): Development and Psychometric Qualities in a Large Community Sample

**DOI:** 10.3390/healthcare12151527

**Published:** 2024-07-31

**Authors:** Sarah Tol, Marieke E. Timmerman, Alina Goltermann, Joost Heutink, Gera A. de Haan

**Affiliations:** 1 Department of Clinical and Developmental Neuropsychology, University of Groningen, Grote Kruisstraat 2/1, 9712 TS Groningen, The Netherlandsj.h.c.heutink@rug.nl (J.H.); g.a.de.haan@rug.nl (G.A.d.H.); 2 Department of Psychometrics and Statistics, University of Groningen, Grote Kruisstraat 2/1, 9712 TS Groningen, The Netherlands; m.e.timmerman@rug.nl; 3 Royal Dutch Visio, Centre of Expertise for Blind and Partially Sighted People, Amersfoortsestraatweg 180, 1272 RR Huizen, The Netherlands

**Keywords:** reading, self-report questionnaire, homonymous visual field defects, hemianopia, bifactor model, validity, reliability, rehabilitation

## Abstract

The ability to read is important for daily life functioning. Individuals with homonymous visual field defects (iwHs) after brain injury experience frequent reading difficulties. The current study presents a novel self-report questionnaire aimed at measuring the wide variety of reading difficulties iwHs can experience: the Hemianopia Reading Questionnaire (HRQ). The 24-item HRQ was developed with help from clinical experts and experts by experience and was inspired by existing reading questionnaires for adults. The three tested subscales of the HRQ assess the relationship to reading, reading skills and daily life functional reading. The factor structure, reliability, convergent validity and divergent validity were examined in a large community sample (i.e., individuals without homonymous visual field defects) with a comparable distribution of age, gender and level of education to those who have suffered a stroke (N = 998). Two competing hypothesized models were tested and a good fit was found for a three-bifactor model of the HRQ. The reliability of the three subscales was found to be good (ω range 0.93–0.99), as well as the convergent and divergent validity (9 out of 12 Spearman’s correlations, according to expectations). The results support further use of the HRQ in iwHs, especially in the context of reading rehabilitation. Suggestions for clinical and scientific use and future psychometric research on the HRQ are provided.

## 1. Introduction

Reading difficulties are very prevalent in individuals with homonymous visual field defects (HVFDs) [1,2], a common consequence of brain damage [3]. Because being able to read well is so important, and entails much more than the reading speed alone, we developed a questionnaire that aims to measure multiple aspects of reading: the Hemianopia Reading Questionnaire (HRQ). In this article, we discuss the development and psychometric qualities of this questionnaire.

Reading is an important aspect of daily life for many people. A large amount of information from educational, professional and social situations is transferred by written text. Furthermore, studies have shown that leisure reading is associated with academic, cognitive and health benefits [4], better long-term social cognition and social engagement [5,6], expressing one’s own individuality from other individuals [7] and better mood-related mental health [8]. Readers have indicated that leisure reading serves multiple educational purposes, for example, in learning about other countries, cultures and history, or gaining new perspectives [9]. A scoping review by Boey and colleagues [10] has shown that reading difficulties in older adults with low vision were associated with self-reported decreased social and leisure activities and activities of daily living since the low vision began, in comparison to control participants. Among older adults with low vision, the goal of ‘reading the newspaper’ was the most mentioned targeted vision function goal [11]. In sum, reading is an important aspect of daily life, and reading impairments hold the potential for widespread negative consequences for individuals experiencing them.

There are several ways to measure an individual’s reading abilities. In a clinical or scientific setting, reading is often measured by calculating the reading speed (and to a lesser extent, reading acuity and reading accuracy) [12,13]. However, other aspects of reading also hold importance, such as reading endurance and comprehension [12]. One way of assessing multiple aspects of reading (e.g., speed, comprehension, pleasure) is with a self-report reading questionnaire [14,15,16]. Multiple studies have shown positive correlations between task performance and self-report measures of reading in the majority of adult samples [15,17,18,19]. Self-report reading questionnaires can provide a more differentiated assessment of reading compared to the measurement of reading speed alone because they have the capacity to address different aspects of reading. To the best of our knowledge, there are three reading questionnaires available for adults with sufficient psychometric qualities. The Adult Reading History Questionnaire (ARHQ) is a screening questionnaire for reading disabilities in adults, taking into account personal reading history and current reading habits [15]. Similarly, the ATLAS was developed as a screening tool for reading and writing difficulties in adults [14]. Finally, the Adult Reading Motivation Scale (ARMS) is a measure of motivation for reading in adults [16]. Each of these questionnaires have been shown to possess good psychometric qualities [14,15,16,20]. However, the questionnaires were not developed for HVFDs, which can result in specific reading difficulties, as explained in the following paragraph.

HVFDs, also denoted as homonymous hemianopia, is a type of visual disorder that may lead to frequent but unique reading difficulties [2,21,22,23]. HVFDs are a common consequence of acquired brain injury, most often stroke [3]. The nature of HVFDs can differ between individuals, in, for example, size (lost quarter to lost half of the visual field), side (loss of the right or left side of the visual field) and the amount of ‘macular sparing’ in approximately the five most central degrees of the visual field [2,21,22,23]. In all HVFDs, the visual field loss is the same for the left and right eye. Due to not being able to process information from a part of the visual field, individuals with HVFDs (iwHs) experience reading difficulties resulting from not being able to see words or sentences and losing an overview of the text [2,21,22,23]. A resulting disorganized eye movement pattern during reading has often been found. Many (reading) interventions for iwHs have been suggested and studied. Most common are compensatory eye movement (saccadic) interventions and restitutive interventions aiming to stimulate the impaired visual field (for an overview of characteristics and reading outcome measures of interventions, see Tol and colleagues [13], for an overview of the effectiveness of interventions see Maeyama and colleagues and Pollock and colleagues [24,25]). Together with mobility difficulties, impaired reading has been reported as one of the most debilitating effects of HVFDs [1].

Reading difficulties and reading interventions have often been studied in iwHs. In a recent review about reading interventions and reading outcome measures for iwHs, we have made suggestions to include validated reading measures in HVFD intervention research for both task performance and self-report measures of reading, which would also be of interest for clinical practice [13]. There are multiple standardized task performance reading measures for iwHs [26,27,28]. A recent study by Hepworth and colleagues was the first to present a self-report questionnaire on visual impairment due to stroke, including one reading item [29]. However, there is no standardized and validated self-report questionnaire only assessing reading for iwHs. Such a questionnaire could have both scientific and clinical value. Clinically, a reading questionnaire could add valuable information to objective task performance measures of reading (e.g., words per minute), creating a more complete picture of the reading difficulties an iwH experiences. Additionally, such a questionnaire could provide direction to reading interventions by identifying reading-related treatment goals. Finally, a standardized and validated questionnaire could be a valuable tool to assess treatment progress and outcomes.

The current study presents a self-report reading questionnaire for iwHs: the Hemianopia Reading Questionnaire (HRQ). The psychometric qualities of this questionnaire will be assessed in a large community sample. With these data, we hope to establish a solid basis for the further use and investigation of the HRQ in iwHs. We will firstly describe the development process of the HRQ. Secondly, an elaboration will be given on the use of a representative community sample. Subsequently, the factor structure and reliability of the HRQ will be investigated by means of Confirmatory Factor Analysis (CFA) with two competing hypothesized models. Lastly, the convergent and divergent validity of the HRQ will be assessed. The aim is to present a questionnaire that can be used for a single measurement of the relevant aspects of reading for iwHs, as well as to provide pre-intervention and post-intervention variants to enable an assessment of the intervention effectiveness.

## 2. Development of the Hemianopia Reading Questionnaire (HRQ)

Our aim was to develop a self-report questionnaire suitable for clinical practice and intervention research that can be administered to adult iwHs. The goal for the questionnaire was to be able to cover a broad spectrum of complaints and wishes for HVFD-induced reading difficulties. The areas of measurement that were considered were reading intervention goals, reading pleasure, the importance of reading, reading difficulties (i.e., including specific HVFD-related reading difficulties and reading endurance) and personal reading history. Additionally, we wanted to take into account a retrospective reflection on the time before the HVFD and create a pre-intervention and post-intervention variant for repeated assessment.

### 2.1. Phase 1: Item Generation

A pool of candidate items was generated partly based on three validated and reliable self-report reading questionnaires for adults: the ARHQ [15,20], the ARMS [16] and the ATLAS questionnaire for reading and writing difficulties in adults [14]. The items were copied or adjusted in terms of phrasing and/or response options to create coherent subscales in the HRQ. The candidate items based on the ARHQ, ARMS and ATLAS were complemented by items created by the authors S.T., G.A.d.H. and J.H. based on clinical experience and relevant literature. Seven initial subscales were created: relationship to reading, reading comprehension, reading skills, reading objects, reading time, reading intervention and reading history (see Table 1). A detailed description of the generation of the items and subscales for the first version of the HRQ can be found in Supplementary Information S1.

### 2.2. Phase 2: Further Development and Expert Face Validity

The HRQ resulting from phase 1 was evaluated by nine visual field disorder experts, consisting of two occupational therapists, two neuropsychologists, one neurolinguist, two clinical physicists and two experts by experience (i.e., iwHs and reading difficulties). In visual rehabilitation centers in the Netherlands, clinical physicists are specialized in visual rehabilitation with regard to the visual system and adaptation to the environment. The experts rated the seven subscales based on the content and HVFD relevance, readability, simplicity, and ambiguity on five-point Likert scales (‘completely disagree’–‘completely agree’). In one-on-one meetings, the experts were asked for further qualitative feedback about phrasing, structure, missed content, whether items should be deleted and the design of the questionnaire. The ratings of the experts on each subscale were used to structure the interview. When an expert rated a subscale as low (score 1 or 2), it was asked why this was rated as low and how to improve this. The feedback of the experts primarily concerned item reduction, removing overlapping items and adding or adjusting items to be more HVFD-specific. After integrating the feedback of the experts, the HRQ consisted of 59 items. These items were recategorized into six subscales. The subscale reading comprehension was deleted as items concerning reading comprehension were included into the reading skills subscale (Table 1).

Two variants of the HRQ were simultaneously created: a pre-intervention and a post-intervention variant. The pre-intervention variant of the HRQ (HRQ-pre) consisted of 59 items, associated with all six subscales. Additionally, for the subscales relationship to reading, reading skills and reading objects, items should be answered twice; once while reflecting on the current time and a second time while reflecting on the time before the HVFD. The post-intervention variant (HRQ-post) consisted of four subscales only, namely, relationship to reading, reading skills, reading time and reading objects, since reading intervention (faith, motivation, goals for intervention) and reading history require being registered only once. In the HRQ-post, the respondent was asked to reflect on the past two weeks while answering the items. The HRQ-post consisted of 27 items (Table 1).

The subscale relationship to reading (HRQ-r) consisted of five items regarding the importance of reading to the individual as well as their self-reported reading efficacy. Example items were ‘Reading is important to me’ and ‘I am a good reader’. Responses regarding whether the respondent agrees with the given statement were on a five-point Likert scale ranging from ‘strongly disagree’ (1) to ‘strongly agree’ (5). The subscale reading skills (HRQ-s) consisted of eight items concerning reading skills deemed important and/or having a high possibility of being impaired in iwHs. In accordance with the example items from phase 1, responses were given on a four-point Likert scale ranging from ‘poorly’ (1) to ‘very well’ (4). The subscale reading objects (HRQ-o) consisted of 11 items, all reflecting sources and objects one could read in daily life, such as a physical book or television subtitles. On the same response scale as the HRQ-s, the respondent was asked to report on how well the reading of these sources went. The respondents could also indicate ‘not applicable’ on this scale. Additionally, open answer opportunities were provided for additional sources. The subscale reading time comprised three open-ended items inquiring about the time spent on obligatory and leisure reading and on reading endurance. The subscale reading intervention consisted of two open-ended items where the respondent could indicate their wishes for potential improvement in specific reading skills and reading objects after a reading intervention. Additionally, two items on the respondents’ faith and motivation for the reading intervention were included in this subscale where the respondent was asked to give these values a grade between 1 and 10. The subscale reading history concerned four items to check for a history of learning and reading difficulties of the respondent.

## 3. On the Use of a Community Sample

We chose for an initial assessment of the HRQ in a large community sample of individuals without HVFDs with a similar distribution of age, level of education and gender as individuals who have suffered an acquired brain injury, primarily stroke (which is the leading cause of HVFDs) [30,31]. This is informative for the nature and quality of the HRQ, and, unlike assessments among iwHs, is feasible to do. In HVFD research embedded in clinical practice, recruiting sufficient participants is challenging [32]. The information gathered from the community sample pertains to the comprehensibility of the questionnaire and to the factor structure. For a factor analysis, researchers agree that the bigger the sample size the better for accurate CFA estimations [33]. Further, substantial sample sizes (>400) are also recommended for precise validity testing [34]. Therefore, the psychometric qualities will be initially studied in a large community sample with similar characteristics as individuals with an acquired brain injury. Consequently, a topic to consider here is measurement invariance (also known as a lack of differential item functioning). If the outcomes of the current study advocate for the further use of the HRQ, measurement invariance should be studied by looking at the factor structure, factor loadings, thresholds and residual variances across the different groups [35]. With regard to the factor structure, we have no reason to assume meaningful differences between the iwHs and a community sample. Some items might load differently, show different thresholds and show different residual variances. This is in regard specifically to the items of the HRQ asking for the current situation because these items concern the situation where the respondent has an HVFD, which is the expected cause for higher dispersion in the HRQ items. The information provided from the analyses in the current study should indicate the justification of and guidance for further research on the HRQ in iwHs.

## 4. Methods

The current study assesses the HRQ subscales HRQ-r, HRQ-s and HRQ-o in a large and representative community sample. The subscales reading time, reading intervention and reading history were not included in the current analysis. Reading time was not included as this scale included three open-ended items. The subscale reading intervention was not included as this scale is specifically designed for the respondent that applied for a reading intervention and thus is non-informative for assessments outside a rehabilitation context. The subscale reading history was not included as this scale was primarily designed for the clinician to check for past reading difficulties, and therefore is not incorporated in the HRQ-post.

To assess the factor structure and reliability of the HRQ in a community sample, the subscales HRQ-r, HRQ-s and HRQ-o were administered. To assess the convergent and divergent validity, four additional questionnaires were administered: the Stokmans reading questionnaire [36], Impact of Vision questionnaire [37,38], Depression, Anxiety and Stress Scale—21 [39,40] and the Behavioral Regulation Index of the Behaviour Rating Inventory of Executive Function—Adult [41,42]. More information on these questionnaires is provided under Section 4.2. (Materials).

### 4.1. Participants and Procedure

This cross-sectional study was performed in a community sample of Dutch adults without a reported history of neurological pathology. The distribution of age, level of education and gender in a quota of 1100 participants (see Supplementary Table S1) was determined based on information on individuals who have primarily suffered a stroke or other acquired brain injury from a large multicenter trial in Dutch rehabilitation centers (N = 502) [43] and openly available data on stroke survivors from the Dutch Central Statistics Office (CBS). Participants were recruited via Panel Inzicht, a Dutch online research panel focused on online quantitative data collection, where participants are provided monetary compensation for participation. Data collection ran between March and June of 2022. Completion of the online survey was estimated to take around 25–30 min and ran with Qualtrics software [44]. This study received ethical approval from the Ethical Committee of Psychology of the University of Groningen (project PSY-2021-S-0504). Respondents were asked to indicate any visual pathology. Presence of self-reported visual pathology was categorized into potentially interfering with reading or not.

### 4.2. Materials

#### 4.2.1. Factor Structure and Psychometric Qualities

**HRQ-post.** For the current study, the subscales HRQ-r, HRQ-s and HRQ-o were used (see Table 1 and Table 2). Since this study focusses on a community sample without self-reported neurological pathology, the HRQ-post was used in which double items about the situation before the HVFD, questions about intervention motivation and questions about reading history are not included. The HRQ-r, HRQ-s and HRQ-o of the HRQ-post altogether consist of 24 items. For the calculation of the convergent and divergent validity of the HRQ, subscale scores were calculated based on the mean item scores. The HRQ was administered in Dutch.

#### 4.2.2. Convergent Validity

**Stokmans Reading Questionnaire.** This questionnaire was developed to evaluate the attitude towards reading and reading behaviour, and was studied in 505 Dutch pupils aged 11–15 [36]. The Stokmans questionnaire comprises different (sub)scales, and the scales concerning reading attitude were taken into account in the current study. The ‘Global reading attitude’ scale is made up of 19 items consisting of two opposing adjectives which reflect the respondents’ opinion on reading. Nine items reflect adjectives relating to hedonistic components of reading (e.g., Interesting—Not interesting), ten items reflect adjectives relating to utilitarian components of reading (e.g., Educational—Not educational). A second subscale of the Stokmans reading questionnaire aims to measure the respondents’ ‘Belief-based attitude’ of reading. This scale includes 14 statements which differentiate the respondents’ motivations for reading into four areas: having fun, empathizing and experiencing a story, education/work performance, learning about life and the world. The Cronbach’s alpha of the six subscales ranges from α = 0.684 to α = 0.936. Scoring is performed using a 5-point Likert scale ranging from ‘Strongly disagree’ (1) to ‘Strongly agree’ (5). The post-intervention version of this questionnaire was used in the current study.

For the purpose of the current study, the means of the aforementioned subscales of the Stokmans reading questionnaire were calculated and summed to one score reflecting reading attitude. This score thus entails both hedonistic and utilitarian components of reading attitude. As the questionnaire by Stokmans has been developed for pupils, the following transformation has been made to one item specifically mentioning school performance: ‘By reading books, I get better grades at school’ into ‘By reading books, I perform better at my education and/or work’. Note, however, that the original questionnaire is in Dutch. Furthermore, values indicating ‘I don’t know’ on the belief-based reading attitude scales were classified as missing values.

**Impact of Vision Impairment Questionnaire (IVI).** The 28-item IVI aims to measure self-reported vision-related quality of life [38,45]. Weih and colleagues validated the IVI in a low-vision sample and found good internal consistency (α = 0.80 to α = 0.96) and test–retest reliability (Guttman’s split-half reliability coefficients 0.73 to 0.94) [38]. In the current study, the ‘Reading and accessing information’ scale of the Dutch version of the IVI was used, which consists of 10 items [37]. The items were scored on a 4-point Likert scale with the options ‘Not at all’ (1), ‘A little’ (2), ‘A fair amount’ (3), and ‘A lot’ (4). Furthermore, each item included the response option ‘Don’t do this for other reasons’, which was labeled as a missing value in the current study. We calculated the average score as a measure of the IVI ‘Reading and accessing information’.

#### 4.2.3. Divergent Validity

**Depression Anxiety Stress Scale—21 (DASS-21).** The DASS-21 is a self-report questionnaire which assesses emotions related to depression, anxiety and stress [40]. These constructs are represented by three corresponding subscales with 7 items each. In a sample of 173 individuals with anxiety and mood disorders, the subscales of the Dutch version of the DASS-21 have shown high reliability (α = 0.89 to α = 0.94), good convergent validity (correlation coefficients from *r =* 0.70 to *r =* 0.82), and good test–retest reliability (*r =* 0.74 to *r =* 0.85) [39]. The items were scored on a 4-point Likert scale with the options ‘Did not apply to me at all’ (0), ‘Applied to me to some degree, or some of the time’ (1), ‘Applied to me to a considerable degree or a good part of time’ (2) and ‘Applied to me very much or most of the time’ (3). The total score is the sum of all items, where a higher score reflects more severe symptoms experienced by the respondent..

**Behaviour Rating Inventory of Executive Function—Adult (BRIEF-A).** The BRIEF-A aims to measure behaviour and emotional expressions of executive dysfunction in everyday life [41,42]. The self-report questionnaire comprises nine subscales with 75 items in total. Additionally, two indices on behavioural regulation and metacognition can be calculated. Items are answered on a 3-point Likert scale with the options ‘Never’ (1), ‘Sometimes’ (2), and ‘Often’ (3). In the current study, the Behavioural Regulation Index (BRI) was used. This scale comprises 30 items and reflects the ability for appropriate regulation of behaviour and emotions [42]. The Dutch version of the BRI has high reliability (α = 0.92) in a sample of the Dutch general population aged 18 to 65 (n = 1600). Moreover, the test–retest reliability of the BRI was also proven to be good, with an intraclass correlation coefficient of *r* = 0.73 (N = 151). In addition to the clinical scales, BRIEF-A contains three validity scales. Two of these scales, the negativity and infrequency scales, were taken into account in the current study. The negativity scale identifies respondents with an unusual negative answering pattern, whereas the infrequency scale identifies respondents whose answers appear atypical (e.g., in random or extreme fashion). In the current study, participants were excluded when they either obtained a score of four or higher on the negativity scale or a score of three or higher on the infrequency scale [41]. This was to ensure a valid response to the included questionnaires.

### 4.3. Statistical Analysis

**Factor Structure of the HRQ.** To evaluate the hypothesized structural properties of the HRQ, we performed CFA in R version 4.2.3 [46] with the package lavaan version 0.6.17 [47]. Two candidate hypothesized models were tested: a 3-factor model, in which all items were loaded onto the three hypothesized subscales, and a 3-bifactor model, in which all items were loaded onto the three hypothesized subscales as well as onto a general factor. Due to the ordinal nature of the items, Diagonally Weighted Least Squares (DWLS) on the polychoric correlation matrix was used to estimate the model parameters [48].

**Data preprocessing.** Responses indicating ‘not applicable’ on the items of the subscale HRQ-o were treated as missing data, as these cannot be easily interpreted on the same scale as the other response categories (i.e., ‘poorly’–‘very well’). Frequency analysis was performed to investigate for how many items ‘not applicable’ was selected on the 11-item scale by each participant. Based on these frequencies, a threshold of 6 was selected. Participants scoring above this threshold (i.e., 7 or more missing items) were excluded for further analysis. For the remaining participants, scores were imputed with the expected maximization technique in SPSS Statistics (version 28). In order to prevent estimation problems (i.e., Heywood cases), for each item, subsequent response categories (e.g., ‘strongly disagree’ and ‘disagree’) were merged until the frequencies within each observed category represented at least 5% of the participants.

**Model Selection.** The following goodness-of-fit statistics were considered: Comparative Fit Index (CFI), Root Mean Square Error of Approximation (RMSEA) and Standardized Root Mean Square Residual (SRMR). Cut-off values as indices of good fit were set at CFI ≥ 0.95, RMSEA ≤ 0.06, and SRMR ≤ 0.08 [49]. If both models met these criteria, then the model with the higher CFI and lower RMSEA in combination with the lower SRMR would be considered the best model. If none of the models met these criteria, modification indices would be inspected and parameters freely estimated in as far as content wise interpretable. The reliability of the subscales was estimated with omega (ω) (equation 11 from [50], based on [51]). In case of a bifactor model, ω reflects the explained variance of the subscale by both the general and the specific factor. The reliability of the HRQ subscales was considered insufficient below 0.70, sufficient between 0.70 and 0.80 and good above 0.80 [52].

**Convergent and Divergent Validity.** To assess the convergent and divergent validity of the HRQ, Spearman’s correlation analysis was performed on the HRQ and Stokmans questionnaire, IVI, DASS-21 and BRIEF-A. We adhered to Cohen’s interpretation of correlation coefficients, indicating values between 0.1 and 0.29 as small, values between 0.3 and 0.49 as medium and values of 0.5 or higher as large [53].

**Table 2:** Demographic characteristics of the study sample.

	Total Sample (N = 998)
Age—M, sd (min-max)	65.3, 13.6 (20–97)
Gender (%)	
Males	546 (54.7%)
Females	450 (45.1%)
Other	2 (0.2%)
Level of education ^1^ (%)	
Low	226 (22.6%)
Middle	405 (40.6%)
High	367 (36.8%)
Self-reported visual pathology that could have an effect on reading ^2^ (%)	126 (13%)

^1^ Dutch classification system recategorized: low: elementary school or lower vocational education (Dutch, e.g., LTS), middle: intermediate vocational education (Dutch: MBO/MULO), high: higher vocational education/university of applied science (Dutch: HBO/VHMO), university [54]. ^2^ See Supplementary Table S5.

To assess the convergent validity, the HRQ subscale means and total score were correlated with the Stokmans reading attitude measure. As the Stokmans questionnaire items reflect reading attitude, whereas the HRQ is hypothesized to assess a broader reflection of both reading attitude and reading performance, we expected medium to large correlations between these measures. Specifically, between the HRQ-r scale and the Stokmans reading attitude measure we expected a large correlation, due to the strongly overlapping constructs. With the HRQ-s and HRQ-o, medium correlations were expected with the Stokmans reading attitude measure. Additionally, we assessed the correlation between the IVI ‘Reading and accessing information’ sum score and the HRQ measures as indicators of convergent validity. Since the IVI subscale is a measure of the impact of vision on activities correlated to near and distance vision including (but not exclusively) reading, we expected medium correlations with the HRQ subscales. Furthermore, we expected correlations between the HRQ and Stokmans measures of larger values than those between the HRQ and IVI, since the Stokmans reading attitude measure concerns reading attitude only, whereas the IVI covers reading as well as other vision-related activities.

To assess the divergent validity of the HRQ, HRQ measures were correlated with the DASS-21 total score and BRI measure of the BRIEF-A. We expected small correlations as the HRQ, DASS-21 and BRI are supposed to measure different constructs. Additionally, we expected the correlations of divergent validity to be of smaller values than those of convergent validity, i.e., Stokmans (medium–large correlations) > IVI (medium correlations) > DASS-21/BRI (small–medium correlations).

## 5. Results

### 5.1. Participants

Before reaching the target of 1100 participants meeting the inclusion criteria, a total of 1944 participants were considered for inclusion. A total of 1100 was selected as the initial goal to ensure a large enough sample size after the potential removal of participants during data cleaning, e.g., due to missing data. Of the 1944, 844 participants could not be included due to the following reasons: not signing the informed consent form (N = 343), age below 18 (N = 27), self-reported history of neurological pathology (N = 195), exact age was not reported (N = 116), and no completion of the survey (N = 163). Of the 1100 participants, 88 had to be excluded due to unusual scores on the negativity scale (N = 4) or infrequency scale (N = 84) of the BRIEF-A. The frequency analysis showed that 14 of the 1012 participants had answered ‘not applicable’ (i.e., treated as missing data) on the HRQ-o items seven or more times. These participants were excluded from further analysis. For the remaining 998 participants, missing responses to the HRQ-o were imputed. Furthermore, responses to items were merged until the set value of 5% of the sample response distribution for every response was reached. For the HRQ-r, the responses ‘strongly disagree’ and ‘disagree’ were merged for items r2, r4 and r5. The responses ‘strongly disagree’, ‘disagree’ and ‘don’t agree or disagree’ were merged for items r1 and r3. Within the subscale HRQ-s, the response categories ‘poorly’ and ‘not well’ were merged for items s7, s12 and s13. In the subscale HRQ-o, this was performed for item o8. The response categories ‘poorly’, ‘not well’ and ‘well’ were merged for items s6 and s8–s11 within the subscale HRQ-s. Within HRQ-o, this was performed with items o1–o7 and o9–o11. The frequencies of the raw and aggregated responses can be found in Supplementary Tables S2–S4. An overview of the demographic characteristics of the study sample can be found in Table 2.

### 5.2. Factor Structure and Reliability of the HRQ

The fit measures for the two tested models of the HRQ can be found in Table 3. The three-bifactor model meets the criteria set of good fit, while the three-factor model does not due to its RMSEA value > 0.06. Therefore, the three-bifactor model of the HRQ was retained. This model is displayed in Supplementary Figure S1. An overview of the factor loadings and explained variance by the general and specific factors in the items is provided in Table 4. Note that three items (s7, s12 and s13) load negatively on the specific factor and the other items load positively on the specific factor. The reliability of the subscales can be classified as good (HRQ-r: ω = 0.93, HRQ-s: ω = 0.98, HRQ-o ω = 0.99).

### 5.3. Convergent and Divergent Validity

The correlations between the HRQ measures, Stokmans questionnaire, IVI, DASS-21 and BRI can be found in Table 5. Unfortunately, one item of the BRI (i.e., item 72) was not included in our online survey. Therefore, we calculated the BRI according to the manual of the BRIEF-A, but without this item. It can be deduced from Table 5 that most of the correlations showed effect sizes according to our expectations. According to our expectations, the largest correlation was found between the Stokmans reading attitude questionnaire and the HRQ-s, which is a large effect size. The relationship between the Stokmans questionnaire and HRQ-o was lower than expected (small instead of medium to large). The correlations between the IVI ‘Reading and accessing information’ scale and HRQ scales were small, where medium effect sizes were expected. This with the exception of the relationship between the IVI and HRQ-o, which shows a medium effect size. Interestingly, the relationship between the IVI ‘Reading and accessing information’ scale with the HRQ-o was found to be larger than the relationship of that same scale with the Stokmans reading attitude questionnaire. All the effect sizes between the DASS-21, BRI and HRQ scales were small, according to our expectations.

## 6. Discussion

The aim of the current study was to develop a self-report questionnaire on a wide spectrum of reading aspects for iwHs (the HRQ) and to investigate the factor structure, reliability and construct (convergent and divergent) validity of this questionnaire in a large community sample with a similar distribution of age, gender and level of education compared to stroke survivors. Two variants of the HRQ were developed: the HRQ-pre is designed for assessment before the start of an intervention, whereas the HRQ-post is suitable for assessment during and after an intervention. Both variants can also be used as a single measurement, depending on the aim of the assessment. Items from three subscales of the HRQ-post were investigated in the current study, which are the relationship to reading (HRQ-r), reading skills (HRQ-s) and reading objects (HRQ-o). A good fit for a bifactor model with one general ‘reading’ factor and three specific factors was found. This supports the use of the three predefined subscales. The reliability of the subscales is good. The validity testing indicated mostly expected effect sizes, indicating the good construct validity of the HRQ [55].

### 6.1. Factor Structure and Reliability

A good fit for the three-bifactor model of the HRQ was found in the community sample. This indicates that there is a general factor, ‘reading’, that represents a part of the covariance of the items of the HRQ, next to a specific (subscale) factor that accounts for the common variance of the items within that subscale [50]. We observed that the items of the HRQ-s especially load high onto the general factor (Table 4), which could indicate that the general factor ‘reading’ is particularly represented by reading skills. A similar observation for a subscale of a questionnaire used to assess psychological problems was made by Smits and colleagues [56]. Following their reasoning, we correlated the HRQ subscales with each other and found higher correlations between the HRQ-s and other subscales (with HRQ-r: *r_s_* = 0.656, *p* < 0.001, with HRQ-o: *r_s_* = 0.620, *p* < 0.001) than between the remaining subscales and each other (*r_s_* = 0.471, *p* < 0.001). This supports the notion that the general factor ‘reading’ primarily reflects reading skills.

An additional notable finding is the contrast effect that was observed within the HRQ-s, where three items (s7, fast reading; s12, reading for a long time without fatigue; and s13, remembering what is read) load negatively on the specific factor in contrast to the remaining items loading positively. A possible explanation for this could be that the contrast effect indicates the existence of two separate subcomponents being represented within the HRQ-s. Potentially, a distinction could be made between basic reading skills such as being able to see the text and orientation within the text and cognitive, or advanced, reading skills such as understanding and remembering what is read. Whether the contrast effect will be observed in a clinical sample remains to be explored. We elaborate on this point under Section 6.4. (Implications and Future Suggestions). Note also that the contrast effect is only observed within the specific factor and not the general factor. Therefore, at this moment, we argue to not divide the HRQ-s into two subscales, and certainly not to reverse items s7, s12 and s13 when calculating a mean score for the HRQ-s. In addition to the methodological argument, these actions would be data-driven and would reflect to a lesser extent the intended functional and clinical use of the HRQ.

The reliability indices for the HRQ subscales (ω) were all values above 0.9. According to Reise [50], this indicates that the subscale scores predominantly reflect a common single factor. Though the seemingly high overlap in constructs between the subscales and the HRQ-s is primarily represented in the general composite factor, the use of subscale scores is more clinically relevant than using a single total scale, and this is justified by our analyses. Additionally, in a qualitative manner, it could be interesting to even monitor the change in specific items, as these can guide intervention goals and monitor intervention progress in a more targeted way.

### 6.2. Convergent and Divergent Validity

Largely, our expectations with regard to the convergent and divergent validity analyses were met, indicating the good construct validity of the HRQ [55]. To the best of our knowledge, there are no Dutch questionnaires for adults, whether or not for HVFDs, aiming to measure reading skills. Therefore, the closest questionnaire with regard to the intended measurement construct available was the Stokmans questionnaire [36]. This questionnaire measures reading attitude, which is related to the respondent’s opinion towards reading and reasons for reading. Indeed, this questionnaire showed the largest correlation with the HRQ-r, which reflects the reading attitude and reading efficacy, combined as aspects of the respondents’ relationship to reading.

It was expected that the Reading and accessing information subscale from the IVI would show medium correlations to the subscales of the HRQ. However, this was only the case for HRQ-o. The IVI subscale is a measure for near vision daily activities (such as reading) that are impacted by vision impairment [38,45]. The current findings suggest that the HRQ-o, more so than the HRQ-r and HRQ-s, reflects functional reading in daily life. Therefore, both clinically and scientifically, this is a valuable subscale to include in the measurement of reading. Including different reading objects to assess the subjective effect of reading interventions in daily life has also recently been implemented in a reading intervention study by Kerkhoff and Kraft [57] but not in many other studies [13].

According to our expectations, the correlations between the HRQ subscales and DASS-21 and BRIEF-A BRI scales were small. This indicates that the self-reported relationship to reading, reading skills and reading objects in daily life do not relate to the self-reported symptoms of depression, anxiety, stress, regulation of behaviour and emotions in the community sample. If these analyses were to be repeated in iwHs, it could be that these correlations would be of higher values, as feelings of worry, frustration, insecurity and dissatisfaction due to an HVFD have been reported in previous research [1,58,59]. However, no consequential differences are expected as the current results indicate the good construct validity of the HRQ.

### 6.3. Limitations

In the current study, we observed very low frequencies of responses indicating low performance in the items (e.g., ‘strongly disagree’ or ‘poorly’). This is not surprising, as the HRQ was developed for iwHs but tested in demographically representative individuals without HVFDs. To be able to reliably assess the factor structure and psychometric qualities of the HRQ, however, this low dispersion could be limiting. Due to the low dispersion, the content validity could be limited when used in individuals without HVFDs [55]. However, in a clinical sample of iwHs, more dispersion is to be expected in both basic reading items as well as items concerning ‘cognitive’ fast reading, reading endurance and remembering what is read [2,3,21,57,60].

One item of the BRIEF-A BRI scale was not present in our dataset, meaning we had to calculate the sum score with 29 items instead of 30. Since we used the BRI with the aim of establishing the divergent validity of the HRQ, and not for, e.g., the classification of the participants, we believe that the risk of faulty conclusions by missing this item in our study is minimal.

Several visual field experts gave input on the development of the HRQ. However, no eye care specialist such as an optometrist or orthoptist was included in this expert group. We cannot exclude the possibility that their comments would have led to different items in the HRQ. Lastly, in this study, the HRQ was administered by means of self-reporting, thus requiring the respondent to read the items. Considering the large sample size we aimed for in the current study, giving participants the option to have the HRQ read aloud would have been a major feasibility obstacle. However, taking into account the ultimate target group of the HRQ (iwHs), future test leaders can consider reading the HRQ out loud while the respondents are able to read along (see HRQ; supplementary materials). This administration method could ensure the respondents’ understanding of the questions and reduce the burden of reporting for the iwH. It could, however, be that the method of administration influences the results of the questionnaire [61].

### 6.4. Implications and Future Suggestions

The three HRQ subscales designed for iwHs were found to fit in a three-bifactor structure in the representative community sample. Taken together with the good construct validity and reliability of the HRQ, the questionnaire can be used in clinical practice and research on reading and interventions for iwHs. Building upon on the findings of the present study, future research on the HRQ needs to focus on confirming the factor structure and psychometric qualities in iwHs. Potentially, in iwHs, subcomponents within the factor structure of the HRQ will be found compared to the three-bifactor model we found in the community sample. Based on previous findings, specifically, differences could be expected with regard to specific items of the HRQ-r and HRQ-s. In the current study, we have hypothesized that the HRQ-r contains elements of the reading attitude and reading efficacy. In the community sample, these items belonged to the same factor, ‘relationship to reading’. It could be possible in a HVFD sample that these two elements reflect different subcomponents since iwHs are expected to experience substantially lower reading efficacy compared to individuals without HVFDs [2,60], whereas not much has been published about the reading attitudes of iwHs compared to individuals without HVFDs. Furthermore, future analyses could explore the added value of item r4 (no reading difficulties) and whether this item should belong in the HRQ-r or HRQ-s, as this item loaded almost exclusively on the general factor ‘reading’, similar to the items belonging to the HRQ-s.

Additionally, we suggest that future research should explore whether the HRQ-s indeed possess two subcomponents in iwHs related to basic (e.g., finding the next line) and cognitive (e.g., remembering what is read) reading skills, and whether the item ‘understanding what is read’ can be classified under basic or cognitive reading skills, as this seemingly advanced reading item did not load as such on the specific factor. Both types of skills have been noted to be impaired in iwHs (for basic reading skills, see, e.g., Zihl [2], Schuett [21], Trauzettel-Klosinksi and Brendler [60] and Horton [62]; for cognitive reading skills, see, e.g., Goodwin [3] and Kerkhoff and Kraft [57]). Additionally, some items would be expected to be especially impaired within HVFD subgroups, such as left-sided HVFDs (item s8, locating the next line), right-sided HVFDs (items s7, fast reading, and s9, finishing reading a line) and iwHs with macular splitting (item s10, perceiving a short word in its entirety). This could uncover a different (sub)factor structure but could also only impact factor loadings, thresholds and/or residual variances.

As far as we know, no studies have reported on the difficulties of reading different objects in iwHs compared to control participants, but following the reasoning mentioned, it is expected that iwHs will report lower scores on all reading objects on the HRQ-o scale compared to individuals without HVFDs. However, no different subcomponents within the HRQ-o are to be expected. In conclusion, in iwHs, future research should assess the factor structure among iwHs, where we would expect the same structure for the HRQ-o and potentially subcomponents within the HRQ-r and HRQ-s subscales. Further, it is warranted to test for measurement invariance in future HVFD samples by looking at the factor structure and, subsequently, factor loadings, thresholds and residual variances across settings [35], as previously mentioned under Section 3 (On the Use of a Community Sample). This is particularly recommended as higher scores by iwHs are expected in most items of the HRQ compared to a community sample.

For further determination of the construct validity of the HRQ in iwHs, we suggest correlation analysis of the HRQ with (1) reading questionnaires for adults. In case of research with English-speaking participants, potential options could be the ARHQ [15], ARMS [16] and ATLAS [14]. (2) Unvalidated reading questionnaires for iwHs (e.g., [57,63]). (3) Reading scales of impact of vision questionnaires such as the National Eye Institute Visual Function Questionnaire (NEI-VFQ-25) [64] and Impact of Visual Impairment questionnaire (IVI) [45,65]. (4) Reading items of the Brain Injury associated Visual Impairment—Impact Questionnaire (BIVI-IQ) [29]. Since the HRQ has also been developed for repeated measures in a reading intervention context, it would also be interesting for future research to explore the reproducibility (i.e., the level of similarity between scores obtained from repeated measures and the extent to which the respondents can be distinguished) and responsiveness (i.e., the ability to detect clinically relevant changes over time) in iwHs [55]. We developed the HRQ with a pre- and post-variant to enable repeated measures within the individual. Therefore, future studies should also investigate the test–retest reliability of the HRQ.

To be able to examine the suggestions given as well as to increase the measurement precision and comparability between studies, we recommend the use of the HRQ in future HVFD reading intervention research. Based on the current results, the HRQ does not seem to be suitable for assessment in individuals without HVFDs due to the low dispersion within items in this sample. However, the established psychometric qualities and factor structure of the HRQ do encourage clinicians and HVFD researchers to use the HRQ as a valid self-report measure of a wide range of reading difficulties in iwHs, suitable for both clinical and scientific contexts. Future research could also keep in mind the potential effects of the administration method (i.e., self-report or read out loud by an administrator) on the HRQ.

## 7. Conclusions

The aim of the present study was to develop and examine a novel self-report questionnaire on reading for iwHs which addresses the wide variety of reading difficulties iwHs can experience. The HRQ possesses good reliability, construct validity and a three-bifactor model based on a large community sample. The studied version of the HRQ consists of three subscales. The subscale HRQ-r measures the respondents’ relationship to reading with items on the reading attitude and reading efficacy. The subscale HRQ-s measures reading skills and the subscale HRQ-o measures daily life functional reading and includes reading objects. Three additional subscales were created for clinical use, but were not analyzed in the current study. Though our data suggest a strong overall composite measure reflecting ‘reading’, we do not advise the usage of a composite measure. The subscales were designed to distinguish specific aspects of reading with the goal to guide and monitor reading interventions for iwHs. Therefore, pre-intervention and post-intervention variants have been developed. With this new instrument, clinicians and researchers are provided with the opportunity to systematically measure different aspects of reading in iwHs. For example, even when an iwH does not improve their reading speed after an intervention, this instrument will be able to measure other potential benefits of reading interventions such as increased understanding, increased reading pleasure and differences in daily reading activities. The use of the HRQ in future HVFD intervention research and clinical practice should also increase the measurement precision and comparability between HVFD intervention studies. Building upon the current findings, a natural progression is to assess the factor structure and psychometric qualities in iwHs, further advocating for the use of the HRQ as a validated self-report reading questionnaire suitable for HVFD interventions.

## Figures and Tables

**Table 1:** Development of the HRQ; overview of subscales and items.

HRQ Subscales (N items)		
Phase 1	Phase 2	
	HRQ-Pre	HRQ-Post
Relationship to reading (9)	Relationship to reading (5 items for 2 situations ^2^)	Relationship to reading; HRQ-r (5)
Reading comprehension (4)		
Reading skills (4 items for 5 situations ^1^)	Reading skills (8 items for 2 situations ^2^)	Reading skills; HRQ-s (8)
Reading objects (12 items for 5 situations ^1^)	Reading objects (11 items for 2 situations ^2^)	Reading objects; HRQ-o (11)
Reading time (3)	Reading time (3)	Reading time (3)
Reading intervention (4)	Reading intervention (4)	
Reading history (5)	Reading history (4)	

HRQ = Hemianopia Reading Questionnaire, HRQ-pre = HRQ pre-intervention variant, HRQ-post = HRQ post-intervention variant. ^1^ For these items, the respondent is asked to rate the item in 5 different situations: (1) How well did this go before the visual field defect? (2) How well did this go in the past two weeks? (3) How important is this in your daily life? (4) How satisfied are you with this at the moment? And, (5) how strong do you feel about wanting to improve this? ^2^ For these items, the respondent is asked to rate the item in 2 different situations: (1) while keeping the past two weeks in mind, and (2) while keeping the time before the visual field defect in mind.

**Table 3:** Fit measures for the competing models of the Hemianopia Reading Questionnaire.

Model	χ2	*df*	CFI	RMSEA (90% CI)	SRMR
3-factor	2114.053	249	0.996	0.087 (0.083–0.090)	0.068
3-bifactor	1012.206	228	0.998	0.059 (0.055–0.062)	0.047

**Table 4:** Factor loadings and explained variance of items of the Hemianopia Reading Questionnaire 3-bifactor model.

Scale	Item	Short Description	Factor Loading		Explained Variance in Item		
			General Factor	Specific Factor	By General Factor	By Specific Factor	By All Factors
Relationship to reading	r1	Being a good reader	0.723	0.450	52%	20%	73%
	r2	Importance of reading	0.562	0.762	32%	58%	90%
	r3	Positive attitude towards reading	0.669	0.618	45%	38%	83%
	r4	No reading difficulties	0.702	0.123	49%	2%	51%
	r5	Love reading	0.556	0.694	31%	48%	79%
Reading skills	s6	Understanding	0.889	0.093	79%	1%	80%
	s7	Fast reading	0.794	−0.063	63%	0.4%	63%
	s8	Locating next line	0.949	0.258	90%	7%	97%
	s9	Finishing a line	0.960	0.255	92%	7%	99%
	s10	Perceiving a short word	0.970	0.202	94%	4%	98%
	s11	Perceiving a long word	0.973	0.180	95%	3%	98%
	s12	Reading for long without fatigue	0.884	−0.315	78%	10%	88%
	s13	Remembering	0.793	−0.141	63%	2%	65%
Reading objects	o1	Physical book	0.784	0.478	61%	23%	84%
	o2	Physical newspaper	0.751	0.579	56%	34%	90%
	o3	Physical magazine	0.790	0.572	62%	33%	95%
	o4	Subtitles	0.648	0.625	42%	39%	81%
	o5	Smartphone	0.604	0.671	36%	45%	82%
	o6	Tablet/e-reader	0.666	0.716	44%	51%	96%
	o7	Laptop/computer	0.628	0.734	39%	54%	93%
	o8	Package leaflets	0.519	0.466	27%	22%	49%
	o9	Traffic signs	0.610	0.745	37%	56%	93%
	o10	Public transport information signs	0.623	0.750	39%	56%	95%
	o11	Letters/mail	0.708	0.626	50%	39%	89%

**Table 5:** Spearman correlations between the HRQ, Stokmans reading questionnaire, IVI, DASS-21 and BRI.

	HRQ-r	HRQ-s	HRQ-o
Stokmans questionnaire reading attitude (N = 967)	**0.604**	**0.382**	0.288
IVI Reading and accessing information mean score (N = 986)	−0.168	−0.286	**−** **0.354**
DASS-21—total score (N = 998)	**−** **0.172**	**−** **0.271**	**−0.290**
BRIEF-A—Behavioural Regulation Index (N = 998)	**−** **0.132**	**−** **0.209**	**−** **0.225**

Notes. All correlations had a *p*-value <=.01. Bolded correlations were according to expectations. HRQ = Hemianopia Reading Questionnaire, HRQ-r = HRQ relationship to reading subscale, HRQ-s = HRQ reading skills subscale, HRQ-o = HRQ reading objects subscale, IVI = Impact of Vision questionnaire, DASS-21 = Depression Anxiety Stress Scale 21-item, BRIEF-A = Behaviour Rating Inventory of Executive Function, Adult.

## Data Availability

The original contributions presented in the study are included in the article/supplementary material. Further inquiries can be directed to the corresponding author/s.

## Supplementary Materials

The following supporting information can be downloaded at https://www.mdpi.com/article/10.3390/healthcare12151527/s1, Table S1: Quota distribution for the Hemianopia Reading Questionnaire data collection; Table S2: Response frequencies of the raw and aggregated scores on the Hemianopia Reading Questionnaire—Relationship to reading subscale (HRQ-r); Table S3: Response frequencies of the raw and aggregated scores on the Hemianopia Reading Questionnaire—Reading skills (HRQ-s); Table S4: Response frequencies of the raw and aggregated scores on the Hemianopia Reading Questionnaire—Reading objects (HRQ-o); Table S5: Frequencies of self-reported visual pathology that could have an effect on reading; Figure S1: 3-bifactor model of the Hemianopia Reading Questionnaire.
