# Supplementary material for: The Hemianopia Reading Questionnaire (HRQ): Development and Psychometric Qualities in a Large Community Sample
Source: Healthcare (Basel). 2024 Jul 31;12(15):1527. doi: 10.3390/healthcare12151527 (PMC11311558; doi:10.3390/healthcare12151527)
Supplement: Supplementary file 1 [file healthcare-12-01527-s001.zip › Questionnaire_HRQ_post_ENG.pdf]

Hemianopia Reading Questionnaire – Post–intervention

To be filled in by the researcher

Date: ………

Participant code: ……………………………………………………………………………………………

This questionnaire can also be filled in by the participant alone or by the researcher together with the participant. In this case, the researcher reads the questions out loud while the participant reads along.

Instructions

This questionnaire will inquire about your experiences with everyday reading. The questionnaire consists of 5 pages. For each question, please indicate which answer lines up best with your own experience. If you have any questions, please feel free to ask the researcher. Make sure to read the explanation of the relevant section before you start answering the questions. If you wear glasses or contact lenses for reading purposes, assume you are wearing them in the scenarios of the questions.

Assessment of own reading

Listed below, you will find a number of statements. Indicate to what extent you agree with the statements.

Answer based on your behaviour in the ***past two weeks***

	strongly disagree	disagree	neither agree nor disagree	agree	strongly agree
1. I am a good reader	1	2	3	4	5
2. Reading is important to me	1	2	3	4	5
3. I have a positive attitude towards reading	1	2	3	4	5
4. I experience no difficulty reading	1	2	3	4	5
5. I love reading	1	2	3	4	5

How did you fare with the following skills in the ***past two weeks?***

	poorly	not well	well	very well
6. Understanding what I read	1	2	3	4
7. Fast reading	1	2	3	4
8. Locating the next line	1	2	3	4
9. Finishing reading a line	1	2	3	4
10. Perceiving a short word in its entirety	1	2	3	4
11. Perceiving a long word in its entirety	1	2	3	4
12. Being able to read for long periods of time without fatigue	1	2	3	4
13. Remembering what I read	1	2	3	4

Reading time

For the three questions listed below, give an estimate of how much time you spend on the activity. Answer based on your behaviour in the ***past month.***

1. How many hours a week do you spend on compulsory reading (for example, for work, studies, personal administration)? …………………. hour(s) per week

2. How many hours per week do you spend reading for fun/leisure (for example, literature, magazines, social media)? …………………. hour(s) per week

3. How many consecutive minutes can you read without getting fatigued? …………………. minutes

<u>What do you tend to read?</u>

Below you will find several different sources and objects that can be read in everyday life. Indicate your experience for each source by circling a number. The scores range from 1 = poor to 4 = very well. If you never use a particular source, you can indicate this by putting a cross in the final column. If this is the case, you do not have to circle a number. After this, you can add sources that you read daily. There is space for this at the bottom of the table. You can write down the source on the dotted line and circle the appropriate rating. Fill in the table based on your activity in the ***past two weeks***.

<u>How did you fare with reading the sources listed below in the ***past two weeks?***</u>

	poorly	not well	well	very well	not applicable
1. Reading a physical book	1	2	3	4	☐ Because of my visual field loss<br>☐ Because of another reason
2. Reading a physical newspaper	1	2	3	4	☐ Because of my visual field loss<br>☐ Because of another reason
3. Reading a physical magazine	1	2	3	4	☐ Because of my visual field loss<br>☐ Because of another reason
4. Reading subtitles	1	2	3	4	☐ Because of my visual field loss<br>☐ Because of another reason
5. Reading from a smartphone	1	2	3	4	☐ Because of my visual field loss<br>☐ Because of another reason
6. Reading from a tablet/e-reader	1	2	3	4	☐ Because of my visual field loss<br>☐ Because of another reason
7. Reading from a laptop/computer	1	2	3	4	☐ Because of my visual field loss<br>☐ Because of another reason

	poorly	not well	well	very well	not applicable
8. Reading package leaflets/packaging	1	2	3	4	☐ Because of my visual field loss<br>☐ Because of another reason
9. Reading traffic signs	1	2	3	4	☐ Because of my visual field loss<br>☐ Because of another reason
10. Reading public transport information boards	1	2	3	4	☐ Because of my visual field loss<br>☐ Because of another reason
11. Reading letters/mail	1	2	3	4	☐ Because of my visual field loss<br>☐ Because of another reason
12………………………………………………	1	2	3	4	☐ Because of my visual field loss<br>☐ Because of another reason
13………………………………………………	1	2	3	4	☐ Because of my visual field loss<br>☐ Because of another reason
14………………………………………………	1	2	3	4	☐ Because of my visual field loss<br>☐ Because of another reason
