# Supplementary material for: The Hemianopia Reading Questionnaire (HRQ): Development and Psychometric Qualities in a Large Community Sample
Source: Healthcare (Basel). 2024 Jul 31;12(15):1527. doi: 10.3390/healthcare12151527 (PMC11311558; doi:10.3390/healthcare12151527)
Supplement: Supplementary file 1 [file healthcare-12-01527-s001.zip › Questionnaire_HRQ_post_NL.pdf]

Hemianopsie Leesvragenlijst – Nameting

In te vullen door de onderzoeker

Datum: ……

Deelnemerscode: ………………………………………………………………………………………………

Deze vragenlijst kan ingevuld worden door de deelnemer zelf of door de onderzoeker samen met de deelnemer. In dit geval leest de onderzoeker de vragen hardop voor terwijl de deelnemer meeleest.

Instructies

In deze vragenlijst wordt er naar uw ervaring met lezen in het dagelijkse leven gevraagd. Deze vragenlijst bestaan uit 5 pagina's. Geef bij elke vraag aan welk antwoord het beste aansluit bij uw ervaring. U kunt bij twijfel altijd de onderzoeker vragen stellen. Lees per onderdeel eerst de uitleg voordat u de vragen beantwoordt. Als u een bril of lenzen heeft voor het lezen, ga er dan bij het beantwoorden van de vragen vanuit dat u deze draagt.

Beoordeling van eigen lezen

Onderstaand vindt u stellingen. Geef voor de stellingen aan in welke mate u het eens bent.

Houdt de ***afgelopen twee weken*** in gedachten bij het antwoorden	sterk oneens	oneens	niet eens of oneens	eens	sterk eens
1. Ik ben een goede lezer	1	2	3	4	5
2. Lezen is belangrijk voor mij	1	2	3	4	5
3. Mijn houding ten opzichte van lezen is positief	1	2	3	4	5
4. Ik ervaar geen moeilijkheden met lezen	1	2	3	4	5
5. Ik houd van lezen	1	2	3	4	5

Hoe goed gingen onderstaande vaardigheden de ***afgelopen twee weken?***

	slecht	niet goed	goed	heel goed
6. Begrijpen wat ik lees	1	2	3	4
7. Snel lezen	1	2	3	4
8. Het vinden van de volgende regel	1	2	3	4
9. Het af lezen van een regel	1	2	3	4
10. Het waarnemen van een kort woord in zijn geheel	1	2	3	4
11. Het waarnemen van een lang woord in zijn geheel	1	2	3	4
12. Onvermoeid door kunnen lezen	1	2	3	4
13. Onthouden wat ik heb gelezen	1	2	3	4

Leestijd

Geef bij onderstaande drie vragen een schatting van hoeveel tijd u hieraan besteedt. Houdt bij het beantwoorden van de vragen **afgelopen maand** in gedachten.

1. Hoeveel uur per week leest u verplicht teksten (Voorbeeld: voor werk, opleiding, eigen administratie)? …………………. uur per week

2. Hoeveel uur per week leest u voor uw plezier/ontspanning (Voorbeeld: literatuur, tijdschrift, sociale media)? …………………. uur per week

3. Hoeveel minuten kan u achter elkaar lezen zonder moe te worden? …………………. minuten

Wat leest u zoal?

Onderstaand vindt u verschillende bronnen die in het dagelijks leven worden gelezen. Geef per bron aan hoe het lezen hiervan gaat door een cijfer te omcirkelen, waarbij 1 = slecht tot 4 = heel goed. Als u bepaalde bronnen nooit leest kunt u dit aangeven door een kruisje te zetten in de laatste kolom en hoeft u geen cijfer te omcirkelen. Hierna kunt u zelf bronnen toevoegen die u in het dagelijks leven gebruikt. Hiervoor is ruimte overgelaten onderaan de tabel. Op de stippellijn kunt u het leesobject schrijven en vervolgens de schaal invullen. Houdt bij het invullen van de tabel ***de afgelopen twee weken*** in gedachten.

Hoe goed ging het lezen van onderstaande bronnen de ***afgelopen twee weken?***

	slecht	niet goed	goed	heel goed	niet van toepassing
1. Papieren boek lezen	1	2	3	4	☐ Door mijn gezichtsvelduitval<br>☐ Door een andere reden
2. Papieren krant lezen	1	2	3	4	☐ Door mijn gezichtsvelduitval<br>☐ Door een andere reden
3. Papieren tijdschrift lezen	1	2	3	4	☐ Door mijn gezichtsvelduitval<br>☐ Door een andere reden
4. Ondertiteling lezen	1	2	3	4	☐ Door mijn gezichtsvelduitval<br>☐ Door een andere reden
5. Van een telefoon lezen	1	2	3	4	☐ Door mijn gezichtsvelduitval<br>☐ Door een andere reden
6. Van een tablet / E-reader lezen	1	2	3	4	☐ Door mijn gezichtsvelduitval<br>☐ Door een andere reden
7. Van een laptop / computer lezen	1	2	3	4	☐ Door mijn gezichtsvelduitval<br>☐ Door een andere reden

	slecht	niet goed	goed	heel goed	niet van toepassing
8. Bijsluiter / verpakkingen lezen	1	2	3	4	☐ Door mijn gezichtsvelduitval<br>☐ Door een andere reden
9. Verkeersborden lezen	1	2	3	4	☐ Door mijn gezichtsvelduitval<br>☐ Door een andere reden
10. Informatieborden openbaar vervoer lezen	1	2	3	4	☐ Door mijn gezichtsvelduitval<br>☐ Door een andere reden
11. Brieven / post lezen	1	2	3	4	☐ Door mijn gezichtsvelduitval<br>☐ Door een andere reden
12…………………………………………………	1	2	3	4	☐ Door mijn gezichtsvelduitval<br>☐ Door een andere reden
13…………………………………………………	1	2	3	4	☐ Door mijn gezichtsvelduitval<br>☐ Door een andere reden
14…………………………………………………	1	2	3	4	☐ Door mijn gezichtsvelduitval<br>☐ Door een andere reden
