# Supplementary material for: The Hemianopia Reading Questionnaire (HRQ): Development and Psychometric Qualities in a Large Community Sample
Source: Healthcare (Basel). 2024 Jul 31;12(15):1527. doi: 10.3390/healthcare12151527 (PMC11311558; doi:10.3390/healthcare12151527)
Supplement: Supplementary file 1 [file healthcare-12-01527-s001.zip › Questionnaire_HRQ_pre_ENG.pdf]

# Hemianopia Reading Questionnaire – Pre-intervention

**To be filled in by the researcher**

**Date: ……………………………………………………………………………………………………………………**

**Participant code: ……………………………………………………………………………………………………………**

*This questionnaire can also be filled in by the participant alone or by the researcher together with the participant. In this case, the researcher reads the questions out loud while the participant reads along.*

**Instructions**

This questionnaire will inquire about your experiences with everyday reading. The questionnaire consists of 5 pages. For each question, please indicate which answer lines up best with your own experience. If you have any questions, please feel free to ask the researcher. Make sure to read the explanation of the relevant section before you start answering the questions. If you wear glasses or contact lenses for reading purposes, assume you are wearing them in the scenarios of the questions.

Assessment of own reading

Listed below, you will find a number of statements. Indicate to what extent you agree with the statements.

| Answer based on your behaviour in the ***past two weeks*** | strongly disagree | disagree | neither agree nor disagree | agree | strongly agree |
|---|---|---|---|---|---|
| 1. I am a good reader | 1 | 2 | 3 | 4 | 5 |
| 2. Reading is important to me | 1 | 2 | 3 | 4 | 5 |
| 3. I have a positive attitude towards reading | 1 | 2 | 3 | 4 | 5 |
| 4. I experience no difficulty reading | 1 | 2 | 3 | 4 | 5 |
| 5. I love reading | 1 | 2 | 3 | 4 | 5 |

| Answer based on your behaviour ***prior to your visual field loss*** | strongly disagree | disagree | neither agree nor disagree | agree | strongly agree |
|---|---|---|---|---|---|
| 6. I was a good reader | 1 | 2 | 3 | 4 | 5 |
| 7. Reading was important to me | 1 | 2 | 3 | 4 | 5 |
| 8. I had a positive attitude towards reading | 1 | 2 | 3 | 4 | 5 |
| 9. I experienced no difficulty reading | 1 | 2 | 3 | 4 | 5 |
| 10. I loved reading | 1 | 2 | 3 | 4 | 5 |

How did you fare with the following skills in the ***past two weeks?***

| | poorly | not well | well | very well |
|---|---|---|---|---|
| 11. Understanding what I read | 1 | 2 | 3 | 4 |
| 12. Fast reading | 1 | 2 | 3 | 4 |
| 13. Locating the next line | 1 | 2 | 3 | 4 |
| 14. Finishing reading a line | 1 | 2 | 3 | 4 |
| 15. Perceiving a short word in its entirety | 1 | 2 | 3 | 4 |
| 16. Perceiving a long word in its entirety | 1 | 2 | 3 | 4 |
| 17. Being able to read for long periods of time without fatigue | 1 | 2 | 3 | 4 |
| 18. Remembering what I read | 1 | 2 | 3 | 4 |

How did you fare with the following skills ***prior to your visual field loss?***

| | poorly | not well | well | very well |
|---|---|---|---|---|
| 19. Understanding what I read | 1 | 2 | 3 | 4 |
| 20. Fast reading | 1 | 2 | 3 | 4 |
| 21. Locating the next line | 1 | 2 | 3 | 4 |
| 22. Finishing reading a line | 1 | 2 | 3 | 4 |
| 23. Perceiving a short word in its entirety | 1 | 2 | 3 | 4 |

| | poorly | not well | well | very well |
|---|---|---|---|---|
| 24. Perceiving a long word in its entirety | 1 | 2 | 3 | 4 |
| 25. Being able to read for long periods of time without fatigue | 1 | 2 | 3 | 4 |
| 26. Remembering what I read | 1 | 2 | 3 | 4 |

20. Which reading skills do you hope to improve by reading training?

a.

b.

c.

Reading time

For the three questions listed below, give an estimate of how much time you spend on the activity. Answer based on your behaviour in the ***past month.***

| | | |
|---|---|---|
| 1. How many hours a week do you spend on compulsory reading (for example, for work, studies, personal administration)? | …………………. | hour(s) per week |
| 2. How many hours per week do you spend reading for fun/leisure (for example, literature, magazines, social media)? | …………………. | hour(s) per week |
| 3. How many consecutive minutes can you read without getting fatigued? | …………………. | minutes |

What do you tend to read?

Below you will find several different sources and objects that can be read in everyday life. Indicate your experience for each source by circling a number. The scores range from 1 = poor to 4 = very well. If you never use a particular source, you can indicate this by putting a cross in the final column. If this is the case, you do not have to circle a number. After this, you can add sources that you read daily. There is space for this at the bottom of the table. You can write down the source on the dotted line and circle the appropriate rating. Fill in the table based on your activity in the ***past two weeks.***

How did you fare with reading sources listed below in the ***past two weeks?***

| | poorly | not well | well | very well | not applicable |
|---|---|---|---|---|---|
| 1. Reading a physical book | 1 | 2 | 3 | 4 | ☐ Because of my visual field loss<br>☐ Because of another reason |
| 2. Reading a physical newspaper | 1 | 2 | 3 | 4 | ☐ Because of my visual field loss<br>☐ Because of another reason |
| 3. Reading a physical magazine | 1 | 2 | 3 | 4 | ☐ Because of my visual field loss<br>☐ Because of another reason |
| 4. Reading subtitles | 1 | 2 | 3 | 4 | ☐ Because of my visual field loss<br>☐ Because of another reason |
| 5. Reading from a smartphone | 1 | 2 | 3 | 4 | ☐ Because of my visual field loss<br>☐ Because of another reason |
| 6. Reading from a tablet/e-reader | 1 | 2 | 3 | 4 | ☐ Because of my visual field loss<br>☐ Because of another reason |
| 7. Reading from a laptop/computer | 1 | 2 | 3 | 4 | ☐ Because of my visual field loss<br>☐ Because of another reason |

| | poorly | not well | well | very well | not applicable |
|---|---|---|---|---|---|
| 8. Reading package leaflets/packaging | 1 | 2 | 3 | 4 | ☐ Because of my visual field loss<br>☐ Because of another reason |
| 9. Reading traffic signs | 1 | 2 | 3 | 4 | ☐ Because of my visual field loss<br>☐ Because of another reason |
| 10. Reading public transport information boards | 1 | 2 | 3 | 4 | ☐ Because of my visual field loss<br>☐ Because of another reason |
| 11. Reading letters/mail | 1 | 2 | 3 | 4 | ☐ Because of my visual field loss<br>☐ Because of another reason |
| 12……………………………………………… | 1 | 2 | 3 | 4 | ☐ Because of my visual field loss<br>☐ Because of another reason |
| 13……………………………………………… | 1 | 2 | 3 | 4 | ☐ Because of my visual field loss<br>☐ Because of another reason |
| 14……………………………………………… | 1 | 2 | 3 | 4 | ☐ Because of my visual field loss<br>☐ Because of another reason |

How did you fare with reading the sources listed below ***prior to your visual field loss?***

| | poorly | not well | well | very well | |
|---|---|---|---|---|---|
| 15. Reading a physical book | 1 | 2 | 3 | 4 | ☐ I never used to read this |
| 16. Reading a physical newspaper | 1 | 2 | 3 | 4 | ☐ I never used to read this |
| 17. Reading a physical magazine | 1 | 2 | 3 | 4 | ☐ I never used to read this |
| 18. Reading subtitles | 1 | 2 | 3 | 4 | ☐ I never used to read this |
| 19. Reading from a smartphone | 1 | 2 | 3 | 4 | ☐ I never used to read this |
| 20. Reading from a tablet/e-reader | 1 | 2 | 3 | 4 | ☐ I never used to read this |
| 21. Reading from a laptop/computer | 1 | 2 | 3 | 4 | ☐ I never used to read this |
| 22. Reading package leaflets/packaging | 1 | 2 | 3 | 4 | ☐ I never used to read this |
| 23. Reading traffic signs | 1 | 2 | 3 | 4 | ☐ I never used to read this |
| 24. Reading public transport information boards | 1 | 2 | 3 | 4 | ☐ I never used to read this |
| 25. Reading letters/mail | 1 | 2 | 3 | 4 | ☐ I never used to read this |
| 26…………………………………………………… | 1 | 2 | 3 | 4 | |
| 27…………………………………………………… | 1 | 2 | 3 | 4 | |
| 28…………………………………………………… | 1 | 2 | 3 | 4 | |

What sources like the ones mentioned above would you like to be able to read better after the reading training?

a.

b.

c.

Reading training

The questions below concern the reading training you will participate in.

| 1. How motivated are you to participate in the scheduled reading training? Assign a rating from 1 to 10. |
|---|
| 1 2 3 4 5 6 7 8 9 10 |

| 2. How much confidence do you have that your reading will improve after the reading training? Assign a rating from 1 to 10. |
|---|
| 1 2 3 4 5 6 7 8 9 10 |

Reading history

Listed below, you will find a number of questions on learning or reading difficulties. Put a cross for *Yes* or *No.* Please note: if you answer No to question 1 or 2, it indicates the end of the questionnaire, and you do not need to proceed with any further questions.

1. Have you ever been assessed for learning on reading difficulties?
   - ☐ Yes
   - ☐ No → End of questionnaire

2. Have any learning or reading difficulties ever been diagnosed?
   - ☐ Yes
   - ☐ No → End of questionnaire

3. Indicate which difficulties have been diagnosed:

   ……………………………………………………………………

4. Have you ever received treatment for these learning or reading difficulties?
   - ☐ Ja
   - ☐ Nee
