# Supplementary material for: The Hemianopia Reading Questionnaire (HRQ): Development and Psychometric Qualities in a Large Community Sample
Source: Healthcare (Basel). 2024 Jul 31;12(15):1527. doi: 10.3390/healthcare12151527 (PMC11311558; doi:10.3390/healthcare12151527)
Supplement: Supplementary file 1 [file healthcare-12-01527-s001.zip › Supplementary materials_HRQ_ST.pdf]

Supplementary material

Supplementary Information S1:

Comprehensive Description of the Item Generation Process in Development Phase 1 of the HRQ.

Our aim was to develop a self-report questionnaire suitable for clinical practice and intervention research, which can be administered on adult iwHs. The goal for the questionnaire was to be able to grasp a broad spectrum of complaints and wishes for intervention, related to the HVFD-induced reading difficulties. Areas of measurement that were considered were: reading intervention goals, reading pleasure, the importance of reading, reading difficulties (i.e. including specific HVFD-related reading difficulties and reading endurance) and reading history. The initial version of the HRQ after item generation in phase 1 consisted of seven subscales. Below, a description is provided with regard of item generation for each of these subscales.

Subscale 1) Relationship to Reading. The goal for this subscale was to include items that relate to the respondents' relationship to reading, such as reading attitude and reading efficacy. The initial subscale was made up of 9 items consisting of statements that should be answered on a 5-point Likert scale ranging from 'strongly disagree' to 'strongly agree'. Four of these items were taken from the ARMS, specifically from the subscales 'reading efficacy' (items 10 and 20) and 'reading as part of the self'(items 5 and 6) (Schutte & Malouff, [16]). Additionally, two items concerning reading attitude and one concerning reading speed from the ARHQ (items 9-11; Lefly & Pennington [15]) were selected and the response options were adjusted to match the wording of the items and response options of the other items within the scale 'relationship to reading'. The same was done with one item from the ATLAS (item 42; Giménez et al. [14]) coming from the subscale 'reading habits'. Lastly, one additional item was added by the authors concerning experienced reading difficulties.

Subscale 2) Reading Comprehension. This subscale included four statements on the respondents' difficulties in reading comprehension, which should be answered on a 5-point Likert scale ranging from 'never' to 'always'. All four items were items from the ATLAS subscale 'current difficulties' that concerned understanding of written text (i.e. items 15-17 and 23).

Subscale 3) Reading Skills. This initial subscale comprised of five questions which each should be answered for five different reading skills. The questions concerned: 1) performance of this skill before the HVFD, 2) performance of this skill in the past two weeks, 3) importance of this skill in daily life, 4) satisfaction with performance on this skill at the moment, and 5) the strength of the wish for improvement of this skill. All questions should be answered on a 4-point Likert scale, for questions 1 and 2 the range was 'bad' to 'very well', for questions 3-5 the range was 'not at all' to 'very much'. The five reading skills were added by the authors but based on earlier mentioned skills in the HRQ, such as fast reading and reading comprehension.

Subscale 4) Reading Objects. Similar to subscale 3, this initial subscale comprised of five questions which each should be answered for different items. These five questions should be answered for 12 different reading objects. These objects were added by the authors, in order to be able to identify potential reading goals of the respondent, as well as to add measurement of reading on a participation-level.

Subscale 5) Reading Time. This subscale was comprised of three open-answer questions concerning the time spent on reading and reading endurance of the respondent. Two items were based on similar questions taken into account in the study of Schutte & Malouff [16] concerning time spent on recreational and required reading. A third item was added by the current authors concerning reading endurance (i.e. time spent on reading before getting fatigued).

Subscale 6) Reading Intervention. Four items were created by the authors regarding a future reading intervention. Two open-answer items concerned goals for reading intervention. The other two items concerned motivation and faith for reading intervention, where respondents were asked to provide a grade between 1 and 10 on these values.

Subscale 7) Reading History. The initial subscale 'reading history' was comprised of five items to gain insight in the existence of pre-HVFD reading difficulties. Two items were copied from the ATLAS subscale 'history of learning difficulties' (items 8 and 10). Additionally, two items from the ARHQ were copied concerning reading performance in elementary school (items 2 and 6). A fifth item was added by the current authors concerning the importance of reading pre-HVFD. All items were answered on a 5-point Likert scale.

Table S1:
Quota distribution for the Hemianopia Reading Questionnaire data collection

Variable	Quota	Final sample
Total group	1100 (100%)	998 (100%)
Gender		
Male	605 (55%)	546 (55%)
Female	495 (45%)	450 (45%)
Other	*45[b] (5%)*	*2 (.02%)*
Age		
18-39	45 (4%)	40 (4%)
40-49	100 (9%)	80 (8%)
50-54	80 (7%)	73 (7%)
55-59	110 (10%)	102 (10%)
60-64	130 (12%)	118 (12%)
65-69	180 (16%)	162 (16%)
70-74	155 (14%)	145 (15%)
75-79	140 (13%)	129 (13%)
80-84	110 (10%)	106 (11%)
85+	50 (5%)	43 (4%)
Level of education[a]		
Low	275 (25%)	226 (23%)
Middle	440 (40%)	403 (40%)
High	385 (35%)	367 (37%)

a. Dutch classification system recategorised: low: elementary school or lower vocational education (Dutch: e.g. LTS), middle: intermediate vocational education (Dutch: MBO/MULO), high: higher vocational education/university of applied science (Dutch: HBO/VHMO), university [54].
b. The quota for gender 'other' was set at 45, which in this case meant that maximally 5% of the participants could have selected this gender in the dataset, taking away maximally 45 individuals from the other two gender response categories.

Table S2

Response frequencies of the raw and aggregated scores on the Hemianopia Reading Questionnaire – Relationship to reading subscale (HRQ-r)

	Raw frequencies					Aggregated frequencies				
Item	Strongly disagree	disagree	Dont agree or disagree	Agree	Strongly agree	Strongly disagree	disagree	Dont agree or disagree	Agree	Strongly agree
r1	9 (0.9%)	38 (3.8%)	145 (14.5%)	559 (56.0%)	247 (24.7)			192 (19.2%)	559 (56.0%)	247 (24.7)
r2	8 (0.8%)	51 (5.1%)	154 (15.4%)	500 (50.1%)	285 (28.6%)		59 (5.9%)	154 (15.4%)	500 (50.1%)	285 (28.6%)
r3	3 (0.3%)	20 (2.0%)	115 (11.5%)	537 (53.8%)	323 (32.4%)			138 (13.8%)	537 (53.8%)	323 (32.4%)
r4	17 (1.7%)	53 (5.3%)	100 (10.0%)	490 (49.1%)	338 (33.9%)		70 (7%)	100 (10.0%)	490 (49.1%)	338 (33.9%)
r5	16 (1.6%)	68 (6.8%)	191 (19.1%)	386 (38.7%)	337 (33.8%)		84 (8.4%)	191 (19.1%)	386 (38.7%)	337 (33.8%)

Table S3

Response frequencies of the raw and aggregated scores on the Hemianopia Reading Questionnaire – Reading skills (HRQ-s)

	Raw frequencies				Aggregated frequencies			
Item	Poorly	Not well	Well	Very well	Poorly	Not well	Well	Very well
s6	3 (0,3%)	20 (2.0%)	584 (58,5%)	391 (39,2%)			607 (60.8%)	391 (39,2%)
s7	8 (0,8%)	148 (14,8%)	607 (60,8%)	235 (23,5%)		156 (15.6%)	607 (60,8%)	235 (23,5%)
s8	2 (0,2%)	20 (2.0%)	582 (58,3%)	394 (39,5%)			604 (60.5%)	394 (39,5%)
s9	1 (0,1%)	17 (1,7%)	589 (59.0%)	391 (39,2%)			607 (60.8%)	391 (39,2%)
s10	0 (0.0%)	14 (1,4%)	584 (58,5%)	400 (40,1%)			598 (59.9%)	400 (40,1%)
s11	2 (0,2%)	33 (3,3%)	584 (58,5%)	379 (38.0%)			619 (62.0%)	379 (38.0%)
s12	11 (1,1%)	134 (13,4%)	557 (55,8%)	296 (29,7%)		145 (14.5%)	557 (55,8%)	296 (29,7%)
s13	12 (1,2%)	103 (10,3%)	633 (63,4%)	250 (25,1%)		115 (11.5%)	633 (63,4%)	250 (25,1%)

Table S4

Response frequencies of the raw and aggregated scores on the Hemianopia Reading Questionnaire – Reading objects (HRQ-o)

	Raw frequencies				Aggregated frequencies			
Item	Poorly	Not well	Well	Very well	Poorly	Not well	Well	Very well
o1	12 (1,2%)	25 (2,5%)	446 (44,7%)	515 (51,6%)			483 (48.4%)	515 (51,6%)
o2	2 (0,2%)	25 (2,5%)	393 (39,4%)	578 (57,9%)			420 (42.1%)	578 (57,9%)
o3	3 (0,3%)	19 (1,9%)	435 (43,6%)	541 (54,2%)			457 (45.8%)	541 (54,2%)
o4	4 (0,4%)	26 (2,6%)	420 (42,1%)	548 (54,9%)			450 (45.1%)	548 (54,9%)
o5	3 (0,3%)	45 (4,5%)	503 (50,4%)	447 (44,8%)			551 (55.2%)	447 (44,8%)
o6	3 (0,3%)	29 (2,9%)	433 (43,4%)	533 (53,4%)			465 (46.6%)	533 (53,4%)
o7	2 (0,2%)	18 (1,8%)	411 (41,2%)	567 (56,8%)			431 (43.2%)	567 (56,8%)
o8	24 (2,4%)	137 (13,7%)	523 (52,4%)	314 (31,5%)		161 (16.1%)	523 (52,4%)	314 (31,5%)
o9	5 (0,5%)	19 (1,9%)	425 (42,6%)	549 (55.0%)			449 (45.0%)	549 (55.0%)
o10	2 (0,2%)	25 (2,5%)	474 (47,5%)	497 (49,8%)			501 (50.2%)	497 (49,8%)
o11	3 (0,3%)	10 (1.0%)	404 (40,5%)	581 (58,2%)			417 (41.8%)	581 (58,2%)

Table S5

Frequencies of self-reported visual pathology that could have an effect on reading

Total sample	998
Total self-reported visual pathology	126
Refractive error that cannot be compensated for with glasses or contact lenses	9
Glaucoma	18
Macular degeneration	13
Cataract	41
Diabetic retinopathy	13
Nystagmus	4
Blepharitis	7
Eye floaters	4
Strabismus	5
Eye stroke	1
Fuch's dystrophy	1
Macular pucker	3
Amblyopia	1
Blindness or loss of one eye	1
Diplopia	2
Dry eyes	2
Damaged conjunctiva	1

Figure S1

3-bifactor model of the Hemianopia Reading Questionnaire

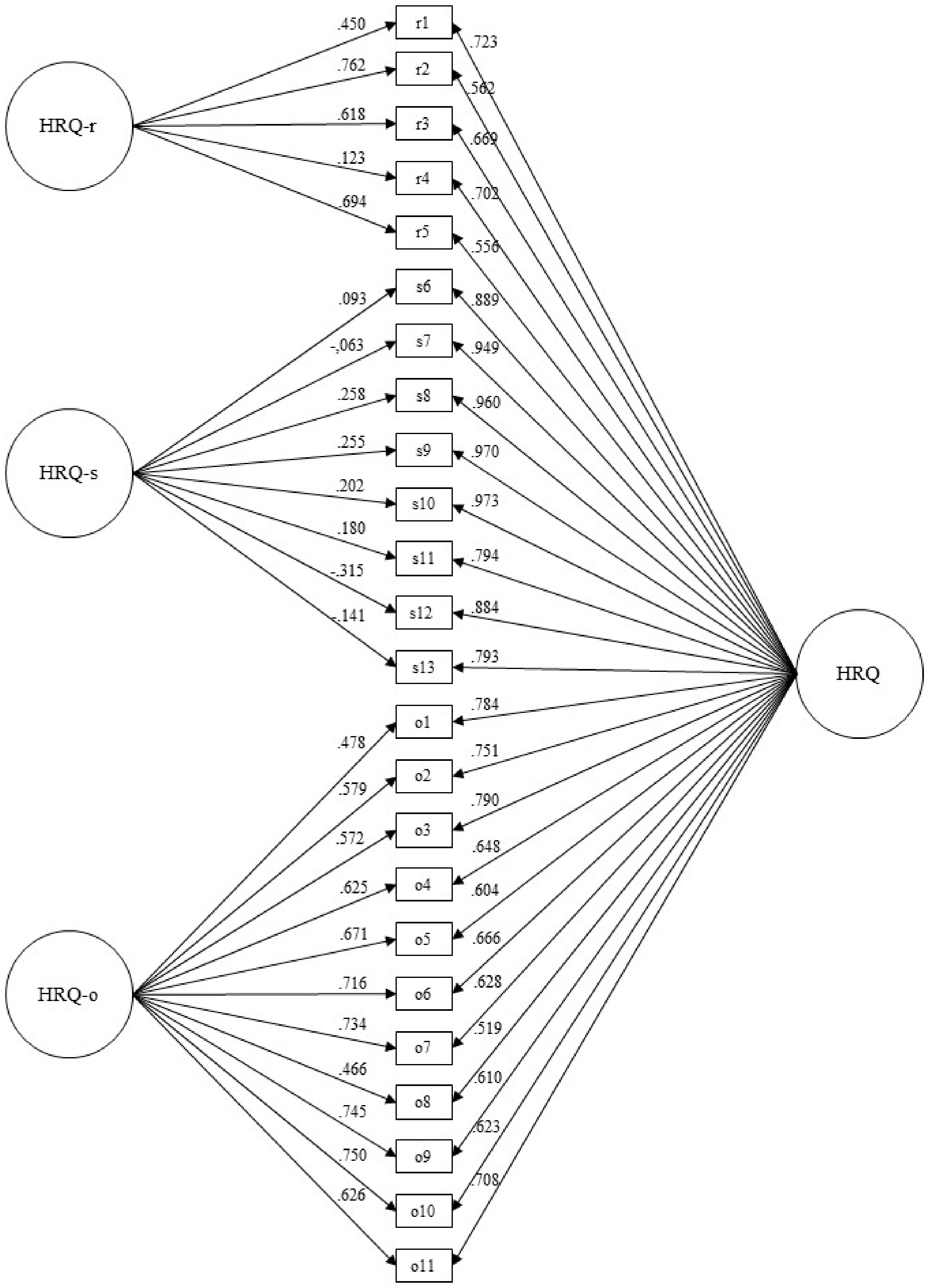

Note. HRQ = Hemianopia Reading Questionnaire, HRQ-r = HRQ relationship to reading subscale, HRQ-s = HRQ reading skills subscale, HRQ-o = HRQ reading objects subscale
